# Mycophenolic Acid Induces the Intestinal Epithelial Barrier Damage through Mitochondrial ROS

**DOI:** 10.1155/2022/4195699

**Published:** 2022-07-05

**Authors:** Yiyun Deng, Zhe Zhang, Hui Yang, Jing Wang, Lijuan Feng, Yong Su, Dujuan Xu

**Affiliations:** ^1^The First Affiliated Hospital of Anhui Medical University, Hefei, Anhui, China; ^2^School of Pharmacy, Anhui Medical University, Hefei, Anhui, China

## Abstract

Mycophenolic acid (MPA) may cause gastrointestinal adverse effects by damaging the intestinal epithelial barrier, the underlying mechanisms remain elusive. Studies have demonstrated that oxidative stress caused by reactive oxygen species (ROS) is linked to tight junction (TJ) proteins and apoptosis, both of which cause abnormalities in intestinal barrier function. Mitochondria, one of the main sources of ROS and abnormally high levels of ROS are linked to mitochondrial dysfunction. The aim of this study was to investigate whether MPA induces intestinal barrier dysfunction through regulation of the mitochondrial ROS. MPA-induced intestinal injury model in Kunming mice and Caco-2 cells. The effect of MPA on Caco-2 cell viability was measured by MTT; tissue diamine oxidase and endotoxin expression were determined by ELISA; expression of total proteins of ZO-1, occludin, Bax, Bcl-2, and mitochondrial proteins of Cytochrome C and Bax was measured by Western blot; and the localization of Cytochrome C with MitoTraker was observed by immunofluorescence staining. Caco-2 cell apoptosis, ROS levels, and mitochondrial membrane potential were detected by flow cytometry, while intramitochondrial ROS levels were observed by MitoSOX fluorescence staining. The results showed that MPA increased intracellular and mitochondrial ROS production to promote oxidative stress and the antioxidant NAC effectively restored ZO-1 and occludin expressions, reduced apoptosis in intestinal epithelial cells. Furthermore, we found that low concentrations of MPA caused mitochondrial damage, induced hyperpolarization of the mitochondrial membrane potential and the translocation of Cytochrome C and Bax proteins from the cytoplasm to the mitochondria. The mitochondrial protectant SS-31 reduces intracellular and intramitochondrial ROS, upregulates TJ, and reduces apoptosis. Our studies suggest that MPA-induced intestinal barrier dysfunction *in vivo* and *in vitro* is mediated, at least in part, by impairing mitochondrial function and promoting oxidative stress.

## 1. Introduction

Mycophenolic acid (MPA) is an inhibitor of hypoxanthine mononucleotide dehydrogenase (IMPDH), which has immunosuppressive effects by inhibiting the guanine nucleotide synthesis pathway and selectively inhibiting T and B lymphocyte proliferation and function [1]. Mycophenolate mofetil (MMF), an ester derivative of MPA, is commonly used as an adjuvant therapy in transplant patients to suppress immune response and reduce graft rejection. However, MPA has a number of negative adverse effects, the most common of which are gastrointestinal adverse effects such as diarrhea and MPA-associated colitis [2]. Watery diarrhea was found to be the most common reason for patients to adjust their MPA dose, which may increase acute rejection and affect graft survival [3, 4]. As a result, determining the mechanism of MPA-induced intestinal adverse reactions is a critical issue that must be addressed as soon as possible.

The intestinal mechanical barrier consists of tight junctions between intestinal epithelial cells. Tight junction disruption or the apoptosis of intestinal epithelial cells can both result in intestinal mechanical barrier dysfunction and increased intestinal permeability, leading to intestinal diseases like diarrhea and enteritis [5, 6]. Tight junction (TJ) proteins are found at the tip of the intestinal epithelium and play an important role in paracellular permeability. They are primarily composed of the transmembrane proteins occludin and claudins and the peripheral protein ZO-1 [7, 8]. The current study found that MPA-associated intestinal toxicity is due to the damage to intestinal epithelial cells. In addition to impairing the expression and distribution of TJ, MPA also induces apoptosis due to its antiproliferative effects [9, 10].

The pathogenesis of many intestinal diseases is known to be influenced by oxidative stress. Numerous studies have found that oxidative stress causes an increase in reactive oxygen species (ROS) production, which leads to apoptosis and barrier damage [11]. Mitochondria are the main source of intracellular ROS, and different toxic stimuli can induce oxidative stress by acting upon them [12]. Oxidative stress usually results in severe oxidative damage to cells or tissues, including disruption of the balance between mitochondria-related apoptosis factors (Bcl-2 family proteins) and promoting the release of Cytochrome C from the mitochondria, thus inducing apoptosis [13]. Several studies have shown that MPA induces mitochondrial dysfunction and promotes the production of ROS in T cells [14]. However, the effect of MPA on intestinal cell mitochondria is unknown; whether MPA affects the function of the intestinal mechanical barrier via ROS remains to be elucidated. Remediation against oxidative stress may be an effective strategy to protect the intestinal barrier from MPA toxicity.

In the present study, we explored the potential mechanisms of MPA-induced intestinal toxicity *in vivo* and *in vitro*. Caco-2 cells are a human colon cancer cell line that freely differentiates to form a monolayer of mature intestinal epithelial cells and are therefore often used as an intestinal barrier model for *in vitro* toxicology studies [15]. We hypothesized that MPA may reduce TJ protein expression and induce apoptosis via mitochondrial ROS.

## 2. Materials and Methods

### 2.1. Reagents

Mycophenolate mofetil capsules were purchased from Roche AG (Basel, Switzerland). MPA (purity ≥ 98%) was purchased from Yuanye Biological Technology Co., Ltd. (Shanghai, China). The reactive oxygen scavenger N-acetyl-117 L-cysteine (NAC), JC-1 probe, TUNEL Apoptosis Detection Kit, Cell Mitochondrial Isolation Kit, and BCA Protein Quantification Kit were purchased from Beyotime Biotechnology (Shanghai, China). The NADPH oxidase inhibitors diphenyleneiodonium chloride (DPI) and mitochondrial protectants D-Arg-Dmt-Lys-Phe-NH2 (SS-31) were purchased from Selleck Chemicals (Houston, Texas, USA). Specific antibodies were purchased from different companies as follows: GAPDH (1 : 10000; Abcam, UK); Bax (1 : 1000; Abcam, UK); ZO-1, Caspase 3, NOX 1, and p22 (1 : 1000; Affinity Bioscience, China); Bcl-2, occludin, and Cytochrome C (1 : 1000; ZENBio, China); and COX IV (1 : 1000; Cell Signaling Technology, USA). The MTT kit was purchased from Sigma Chemical (St. Louis, MO, USA). The flow cytometry apoptosis detection kit was bought from Bestbio Biotechnology Co., Ltd. (Shanghai, China). The ELISA kits were purchased from Jiangsu Feiya Biological Technology Co., Ltd. DHE and H_2_DCFDA fluorescent probes were bought from KeyGEN BioTECH (Nanjing, China). MitoSox-Red fluorescent probes were bought from Invitrogen (Paisley, UK). The SOD and MDA assay kits were bought from Jiancheng Bioengineering Institute (Nanjing, China).

### 2.2. Animals and Treatment

Male Kunming (KM) mice (18–22 g) were purchased from the Animal Experiment Center of Anhui Medical University. They were housed under a 12 h light/dark cycle at room temperature with *ad libitum* access to food and water. Kunming mice were administered MPA intragastrically once daily (500 mg/kg, equivalent to ten times the patient's therapeutic dose) for 21 days. The control mice received the same volume of saline. The mice were sacrificed 24 h after the last treatment, and the ileal tissue and serum were obtained from the animals for subsequent experiments. All animal experiments were reviewed and approved by the Animal Experimentation Ethics Committee of Anhui Medical University.

### 2.3. Cell Culture and Treatment

The human colon adenocarcinoma cell line Caco-2 was kindly provided by Dr. Shuai Song (The First Affiliated Hospital of Anhui Medical University, China). Cells were routinely grown in culture flasks at 37°C with 5% CO_2_ in DMEM (Gibco, MD, USA) supplemented with 10% FBS (Gibco, MD, USA) and 1% streptomycin/penicillin. For MPA experiments, Caco-2 cells were treated with various concentrations of MPA. To explore the mechanism of MPA-induced toxicity, the cells were treated with different concentrations of NAC, DPI, or SS-31.

### 2.4. Histopathological Evaluation

Ileum was fixed in 4% paraformaldehyde and then embedded in paraffin. After sectioning, hematoxylin-eosin (H&E) staining was performed to assess intestinal damage. Morphological changes were assessed by measuring the height of the villi and depth of the crypts under an optical microscope.

### 2.5. Intestinal Permeability

Blood samples were collected from each group, and serum was taken out after centrifugation. According to the manufacturer's instructions, levels of diamine oxidase (DAO) and endotoxin (ET) were detected by the ELISA kit.

### 2.6. Cell Viability Assay

The conventional MTT (3-(4,5-dimethylthiazol-2-yl)-2,5-diphenyltetrazolium bromide) assay was used to determine the toxicity of MPA to Caco-2 cells. Cells were inoculated into 96-well plates and treated with 0–100 *μ*M MPA for 24 h after walling. Each well was incubated with 0.5% MTT solution for 4 h in the dark; then, the medium was discarded, and 150 *μ*L DMSO was added before measuring absorbance at 490 nm using a microplate spectrophotometer (Bio-Tek, USA).

### 2.7. Reactive Oxygen Species (ROS) Measurement

The tissue sections were stained with dihydroethidium (DHE) and observed with a confocal microscope at a wavelength of 530 nm. Cells were treated with 5 *μ*M H_2_DCFDA working solution at 37°C for 20–30 min and immediately detected with flow cytometry (Beckman Coulter, Brea, CA, USA) after washing with PBS. The level of mitochondrial ROS was evaluated by the uptake of MitoSox-Red. After different treatments, cells were stained with 5 *μ*mol/L MitoSox-Red, incubated for 15 min, and then, nuclei were stained with Hoechst 33342 for 10 min. Images were obtained by fluorescent inverted microscopy (OLYMPUS, Japan).

### 2.8. SOD Activity Analysis, MDA Content Determination, and NADPH-OX-1 (NOX 1) Activity Analysis

The ileum tissue sample was evenly ground in saline. The tissue homogenate was centrifuged at 3000 rpm for 15 min, and then, the supernatant was collected. Caco-2 cells were lysed and collected to obtain the cell suspension, which was added to 96-well plates to determine the activities of BCA, SOD, and MDA using assay kits according to the manufacturer's instructions. Also, the levels of NOX 1 were detected by ELISA, according to the manufacturer's instructions.

### 2.9. Immunofluorescence Analysis

The ileum tissue was fixed with 4% paraformaldehyde at 4°C for 24 h and then cryoprotected with 30% sucrose at 4°C overnight. The tissue samples were embedded in tissue (Tek O.C.T) and stored at −80°C. Each frozen section was cut into 5 *μ*m thick frozen sections. After fixing in 4% formaldehyde at room temperature for 15 min and permeabilizing in 0.5% Triton X-100 for 10 min, the cells were blocked with 1% bovine serum albumin for 30 min at 37°C. Subsequently, the cells were incubated with ZO-1 antibody (1 : 250) and occludin antibody (1 : 100) overnight at 4°C. Then, the sections or cells were incubated with the secondary Alexa Fluor 488- (FITC-) or 594- (phalloidin-) conjugated antibodies for 1 h in the dark at room temperature. DAPI was used to dye nuclei. The cells used to analyze the localization of Cytochrome C were added to Mito-Tracker Red CMXRos 15 min before the end of the administration and incubated at 37°C. Then, cells were fixed and blocked as described above, incubated with the Cytochrome C antibody (1 : 200) overnight, and then incubated with FITC-labelled goat anti-mouse IgG at 37°C for 1 hour, before staining the nucleus with DAPI, washing with PBS and observing cells under a confocal microscope (Zeiss, Germany).

### 2.10. TUNEL Assays

TUNEL assays were performed using a TUNEL Apoptosis Detection Kit. Paraffin sections were deparaffinized in xylene, treated with ethanol and distilled water, and incubated with DNase-free proteinase K (20 *μ*g/mL) for 25 min before being washed with PBS 3 times. Then, cells were incubated in endogenous peroxidase strong blocking solution (P0100B) for 20 min at room temperature and washed 3 times with PBS. Next, 50 *μ*L of biotin labelling solution was added to each sample and cells were incubated at 37°C for 60 min in the dark. After adding Streptavidin-HRP working solution and DAB chromogenic solution, the nucleus was stained with hematoxylin staining solution (C0107) and washed 3 times with PBS. TUNEL-positive cells were observed at a magnification of 400x (OLYMPUS, Japan).

### 2.11. Annexin V/FITC Assay

After the cells were digested and collected, they were washed twice with PBS. Cells used to detect apoptosis were resuspended with 400 *μ*L of Annexin V binding solution, treated with Annexin V-FITC, and incubated for 15 min in the dark. Then, PI staining solution was added and cells were incubated in the dark for 5 min. The stained samples were quantified by a flow cytometer (Beckman Coulter, Brea, CA, USA).

### 2.12. Mitochondrial Membrane Potential (Δ*ψ*_m_)

The change in Δ*ψ*_m_ was estimated by the JC-1 probe. At the end of the culture, the treated cells were incubated with JC-1 at 37°C for 20 min. Subsequently, they were washed twice with JC-1 staining buffer and then observed under a fluorescence microscope with excitation/emission (Ex/Em) at 514/529 nm and 585/590 nm or detected with a flow cytometer.

### 2.13. Extraction of Mitochondrial and Cytoplasmic Proteins

Mitochondria and the cytoplasm were separated using a Cell Mitochondrial Isolation Kit. In brief, Caco-2 cells were digested and collected, centrifuged at 200 g for 5 min, and gently resuspended in PBS. The supernatant was discarded after centrifugation at 600 g for 5 min, and the mitochondrial separation reagent was added to an ice bath for 15 min; a homogenizer was used to grind the cells until the number of viable cells reached about 50%. The homogenate was centrifuged at 600 g for 10 min, and the supernatant was centrifuged at 11000 g for 10 min. The resulting supernatant included the cytoplasmic protein. The precipitate was lysed and centrifuged to obtain mitochondrial protein.

### 2.14. Western Blot

Total protein was extracted from mouse ileum tissue and Caco-2 cells. Tissue or cells were lysed with RIPA lysis buffer. Protein samples were loaded and separated by 12% SDS-PAGE and transferred to PVDF membranes (Millipore, Bedford, MA, USA). After blocking with 5% milk for 1 h at room temperature, the membranes were washed three times with TBST and incubated with primary antibodies overnight at 4°C. The membranes were washed and probed with the appropriate secondary peroxidase-conjugated antibody (ZSGBbio, China) for 1 h at 37°C. For all gels, GAPDH was used as the internal standard. The membranes were detected by electrochemiluminescence (Applygen Technologies, Inc. China). Protein bands were quantified with ImageJ software.

### 2.15. Electron Microscope

Tissues were fixed with 2.5% glutaraldehyde for 30 min at room temperature, washed three times with 0.1 M phosphate buffer, fixed for 3 h in 1% osmium fixative, and then washed three more times. They were then dehydrated with graded concentrations of ethanol and different concentrations of acetone; after embedding, tissues were placed in an oven at 37°C to cure overnight, followed by 45°C for 12 h and 60°C for 48 h. Sections were double-stained with 3% uranyl acetate and lead citrate, and the slides were observed using transmission electron microscopy (JEOL, Japan).

### 2.16. qRT-PCR

Total RNA was extracted from the cells by adding TRIzol (Invitrogen, Carlsbad, CA), chloroform, and isopropanol. qRT-PCR was performed using SYBR Reverse Transcription Kit (Vazyme, Nanjing, China) after quantification. The corresponding cDNA and primers were added to the 10 *μ*L system according to the SYBR Green mix (Vazyme) instructions (primer sequences are shown in Table 1). Quantitative real-time PCR was immediately performed using the iCycleriQ Real-Time PCR Detection System. Results are expressed as relative mRNA expression with a blank control normalized to 1.0. GAPDH was used as an internal control. Relative gene expression levels were analyzed by the 2^-*ΔΔ*Ct^ method.

### 2.17. Statistical Analysis

Data were presented by means ± the mean standard deviation (SD) of at least three independent experiments. Social Sciences v.26.0 software (SPSS Inc., Chicago, IL, USA) was used to analyze the data. Multiple comparisons were carried out by one-way (ANOVA) and the independent-sample *t*-test. ^∗^*P* < 0.05 was considered statistically significant (^∗^*P* < 0.05, ^∗∗^*P* < 0.01).

## 3. Results

### 3.1. Effects of MMF on the Body and Intestine of Mice

**Figure 1:**
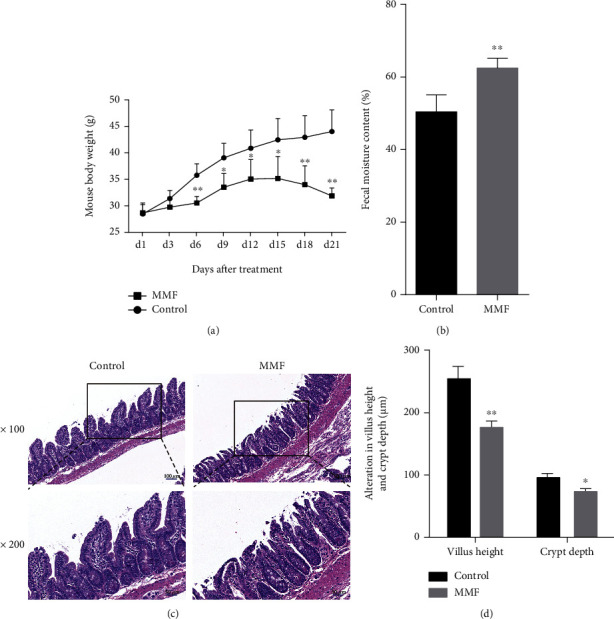
Effect of MMF on the body weight and feces of mice. KM mice were randomly divided into two groups, the control group received saline and the MMF group received MMF (500 mg/kg) for 21 consecutive days, and then analyzed for changes in mice body weight (a) and differences in fecal water content (b). The ileum section was stained with H&E ((c) 100x/200x), and the villus height and crypt depth were measured by persons unrelated to this experiment (d). Data are expressed as the means ± SD, *n* = 6 in each group; ^∗^*P* < 0.05, ^∗∗^*P* < 0.01 significantly different from the control.

In this study, the body weight of mice in the MMF (500 mg/kg) group began to decrease on day 15 (Figure 1(a)) and the fecal water content was significantly higher than that of the control group (^∗∗^*P* < 0.01, Figure 1(b)). The mice were sacrificed at the end of the administration, and the histological examination results are shown in Figure 1(c). In the control group, no obvious intestinal mucosa or intestinal villi injury was observed, while the MMF group showed villi atrophy and structural damage, suggesting that MMF caused intestinal injury. Notably, after measurement, although the height of the villi in the MMF group decreased, the depth of the crypts also decreased (Figure 1(d)).

### 3.2. MMF-Induced Intestinal Barrier Destruction in Mice

**Figure 2:**
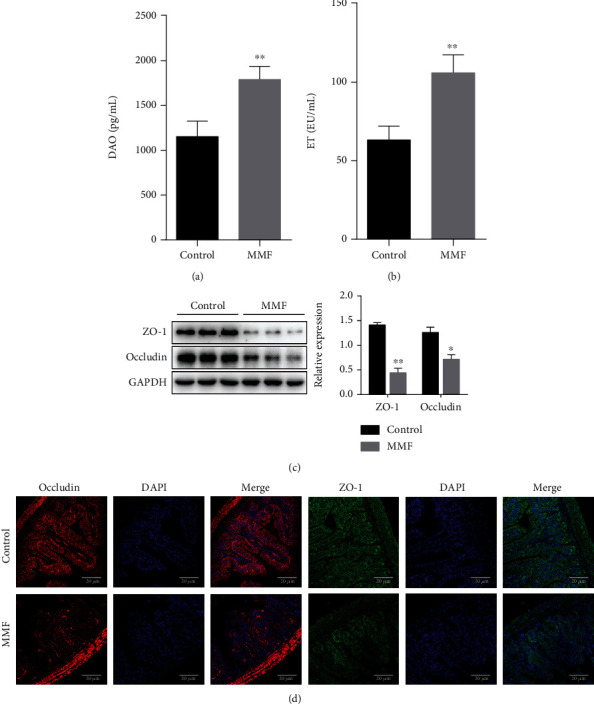
MMF induces intestinal barrier destruction in vivo. KM mice were randomly divided into two groups, the control group received saline and the MMF group received MMF (500 mg/kg) for 21 consecutive days. The level of Serum DAO and ET were detected by ELISA (a, b). The expression of ZO-1 and occludin proteins in the ileum tissue was detected by Western blot (c) and immunofluorescence assay (d). Occludin showed red fluorescence and ZO-1 showed green fluorescence, and DAPI-stained nuclei showed blue fluorescence. Data are expressed as the means ± SD, *n* = 3; ^∗^*P* < 0.05 and ^∗∗^*P* < 0.01 significantly different from the control. The bar represents 20 *μ*m.

Serum diamine oxidase (DAO) activity and endotoxin (ET) levels are important indicators for evaluating the integrity of the intestinal barrier [16]. Compared with the control group, the two indicators in the MMF group were significantly increased (Figures 2(a) and 2(b), ^∗∗^*P* < 0.01), suggesting that MMF increased the intestinal permeability of mice. On the other hand, as an important part of the intestinal mechanical barrier, TJ play a key role in maintaining intestinal function. Therefore, we studied the effect of MMF on the expression of TJ. WB results showed that the expression of ZO-1 and occludin proteins in the MMF group was significantly reduced (Figure 2(c)) and the same results were obtained in immunofluorescence experiments (Figure 2(d)), indicating that the intestinal mechanical barrier was destroyed by MMF.

### 3.3. MMF-Induced Apoptosis in Mice

**Figure 3:**
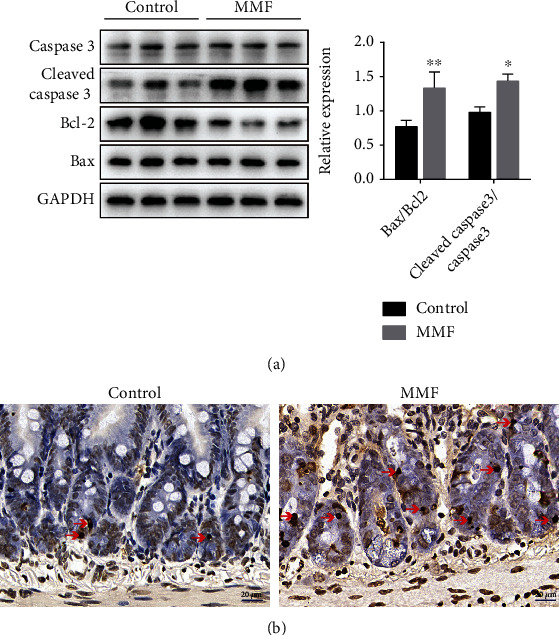
MMF induces apoptosis in vivo. KM mice were randomly divided into two groups, the control group received saline and the MMF group received MMF (500 mg/kg) for 21 consecutive days, and then, the expression of Bax/Bcl-2, cleaved caspase-3/caspase-3 in intestine tissue was detected by Western blot (a). Red arrows indicate that the cells incorporating BrdU are distributed in the crypt (b) (400x). Data are expressed as the means ± SD, *n* = 3; ^∗^*P* < 0.05, ^∗∗^*P* < 0.01 significantly different from the control. The bar represents 20 *μ*m.

Previous reports suggest that MMF may induce apoptosis in intestinal cells. Our results are consistent with previous studies [17]; as shown in Figure 3(a), the ratio of proapoptotic protein Bax to antiapoptotic protein Bcl-2 (^∗∗^*P* < 0.01) and the activation of caspase 3 increased significantly (^∗^*P* < 0.05). Apoptosis in the intestinal tissue was estimated by detecting TUNEL-positive cells in the ileum; our results showed that, compared with the control group, the number of apoptotic cells in the MMF group increased (Figure 3(b)).

### 3.4. MPA Downregulated TJ Expression and Induced Apoptosis in Caco-2 Cells

**Figure 4:**
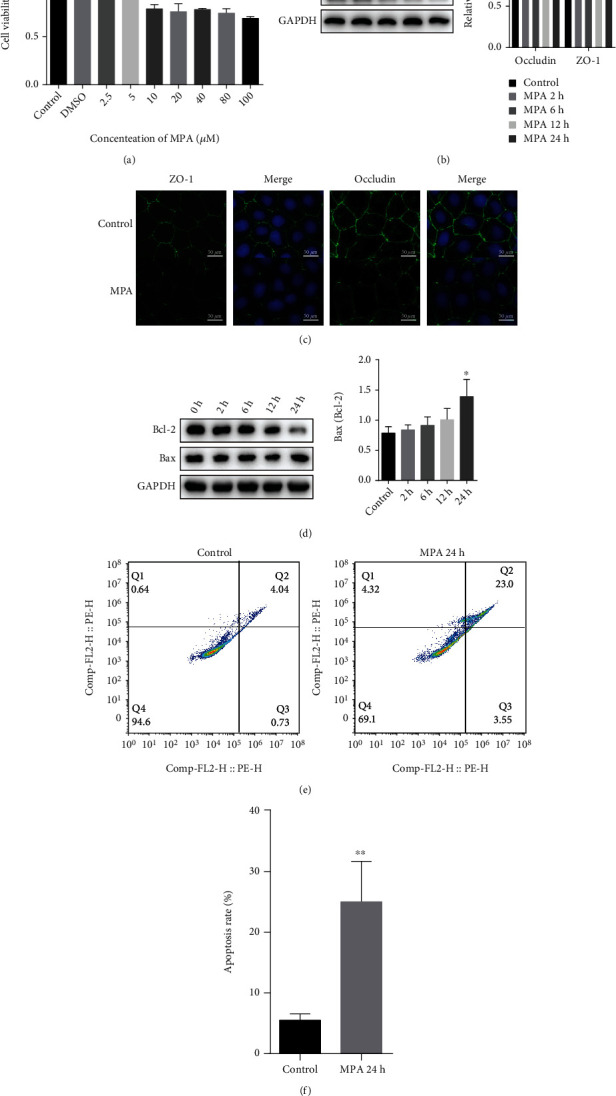
MPA downregulated TJ expression and induced apoptosis in Caco-2 cells. Cytotoxicity was assessed by MTT assay after 24 h of exposure to 0-100 *μ*M MPA (a). Protein expression of occludin and ZO-1 in Caco-2 cells assessed by Western blot (b). Immunofluorescence staining of occludin and ZO-1 in Caco-2 cells (c). Occludin and ZO-1 showed green fluorescence, and DAPI-stained nuclei showed blue fluorescence. Protein expression of Bax/Bcl-2 in Caco-2 cells assessed by Western blot (d). Apoptosis was detected by flow cytometry after membrane linked protein V-FITC/PI staining (e). (f) Quantification of (e). Data are expressed as the means ± SD, *n* = 3; ^∗^*P* < 0.05, ^∗∗^*P* < 0.01 significantly different from the control.

Caco-2 cells were used as a model to investigate the molecular mechanism of MMF-induced intestinal injury. MPA is the main active metabolite of MMF, and we determined the appropriate concentration of MPA for Caco-2 cells by MTT analysis. Caco-2 cells were treated with different concentrations of MPA for 24 h, and the cytotoxicity of MPA increased in a concentration-dependent manner (Figure 4(a)), with 10 *μ*M MPA being used in this study. We evaluated expression of the TJ proteins occludin and ZO-1. Western blot showed that MPA exposure reduced the levels of occludin and ZO-1 proteins in a time-dependent manner, with the effect being most significant at 24 h (Figure 4(b)). Immunofluorescence staining also confirmed the reduced expression of TJ at 24 h (Figure 4(c)). Consistent with *in vivo* experiments, the study showed that the ratio of proapoptotic protein Bax to antiapoptotic protein Bcl-2 increased significantly in Caco-2 cells (^∗^*P* < 0.01) (Figure 4(d)). Apoptosis was then measured by Annexin V-FITC/PI staining. As shown in Figures 4(e) and 4(f), MPA significantly increased the early and late apoptosis rates of Caco-2 cells and reduced the percentage of normal cells at 24 h (^∗∗^*P* < 0.01).

### 3.5. The MPA-Induced Decrease in TJ Expression and Apoptosis Is Mediated by Oxidative Stress in Mouse Intestinal Tissue and Caco-2 Cells

**Figure 5:**
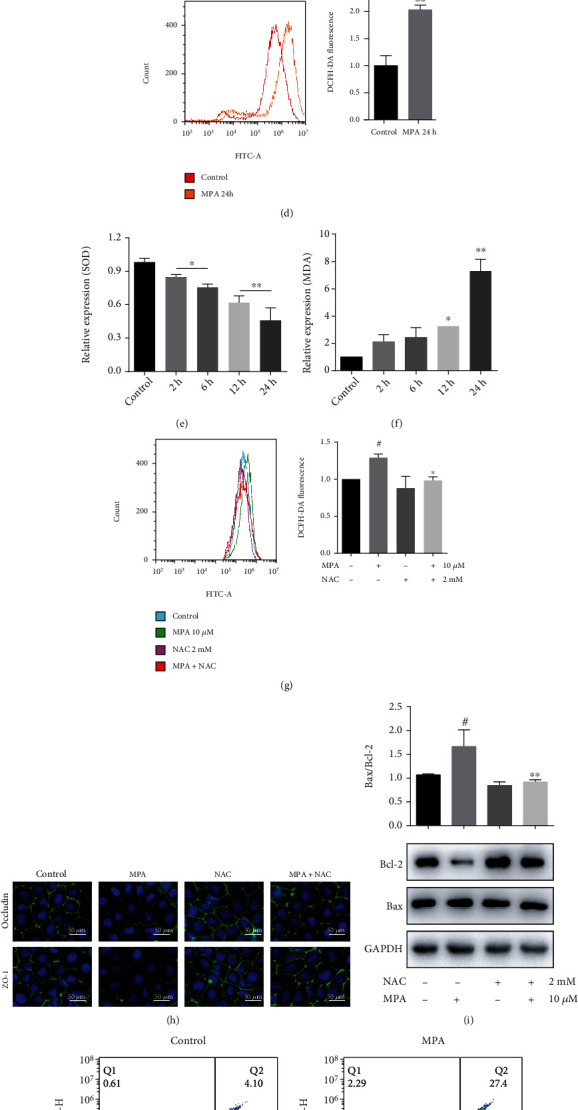
The MPA-induced decrease in TJ expression and apoptosis is mediated by oxidative stress in mouse intestinal tissue and Caco-2 cells. ROS levels (a, d), SOD levels (b, e), and MDA content (c, f) in mice tissues and Caco-2 cells after MPA induction. ROS in mouse tissues showed red fluorescence. After exposure to 2 mM of NAC or 10 *μ*M MPA for 24 h, intracellular ROS levels (g) and apoptosis (j) were detected by flow cytometry, occludin, ZO-1, and Bax/Bcl-2 protein expressions in Caco-2 cells was detected by immunofluorescence (h) or Western blot (i). Occludin and ZO-1 showed green fluorescence, and DAPI-stained nuclei showed blue fluorescence. Dates are expressed as the means ± SD, *n* = 3; ^#^*P* < 0.05, ^##^*P* < 0.01 significantly different from the control. ^∗^*P* < 0.05, ^∗∗^*P* < 0.01 significantly different from the control mycophenolic acid-treated cells.

Many gastrointestinal disorders are caused by an imbalance of the oxidation and antioxidant systems, as well as an increase in ROS. To investigate the mechanism of MPA-induced intestinal injury, we first measured the generation of ROS. The results of DHE staining experiments showed elevated levels of ROS in the intestinal tissues of MMF-treated mice compared with control animals (Figure 5(a)). Next, we tested the activity of SOD and the content of MDA. As shown in Figures 5(b) and 5(c), compared with control animals, MMF significantly reduced SOD activity and increased MDA content in intestinal tissues (^##^*P* < 0.01). Similar results were obtained for Caco-2 cells treated with MPA for 24 h. In Caco-2 cells, intracellular ROS levels were increased in the MPA group compared with the control group after H_2_DCFDA staining (^##^*P* < 0.01, Figure 5(d)) and the SOD activity gradually decreased with increasing time, while the MDA content gradually increased (^#^*P* < 0.05, ^##^*P* < 0.01, Figures 5(e) and 5(f)).

The treatment of Caco-2 cells with 2 mM NAC, a recognized reactive oxygen scavenger, significantly inhibited MPA-induced ROS generation (^∗^*P* < 0.05, Figure 5(g)). In addition, the treatment of Caco-2 cells with NAC restored the expression of occludin and ZO-1 proteins (Figure 5(h)) and reduced apoptosis (^∗∗^*P* < 0.01, Figures 5(i) and 5(j)). And qRT-PCR amplification revealed that occludin and ZO-1 mRNA expressions were significantly increased in NAC-treated cells compared to the MPA group (^∗^*P* < 0.05, Figure S2a). These results suggest that MPA induces oxidative stress *in vivo* and *in vitro* and that the MPA-induced decrease in TJ expression and increase in apoptosis are mediated by ROS.

### 3.6. Effects of MPA on Mitochondria

Reduced nicotinamide adenine dinucleotide phosphate (NADPH) oxidase (NOX) and mitochondria are the main sources of ROS. To investigate the mechanism of MPA-induced ROS elevation, we first examined the protein expression of NOX 1, the most expressed isoform of NADPH oxidase in the intestine, and its ligand p22 by Western blot. However, NOX 1 did not increase in expression after 24 h of MPA treatment and p22 showed a trend of first increasing and then decreasing (Figure S1a). The results of ELISA experiments showed that the enzyme activity of NOX 1 was also not elevated (Figure S1b). qRT-PCR amplification showed that NOX 1 and p22 mRNA expression was significantly reduced compared to the control group. The mRNA expression of the other two isoforms of NADPH oxidase, NOX 4 and NOX 2, and the ligand of NOX 2, p67, were also not upregulated (Figure S2c). We then treated Caco-2 cells with 1 *μ*M or 5 *μ*M NADPH oxidase inhibitor DPI for 24 h. Western blot experiments showed that inhibition of NADPH oxidase at low concentrations did not restore TJ expression or reduce apoptosis, while increasing the inhibitor concentration was toxic to intestinal epithelial cells (Figure S1c, d). qRT-PCR amplification showed similar results (Figure S2b).

**Figure 6:**
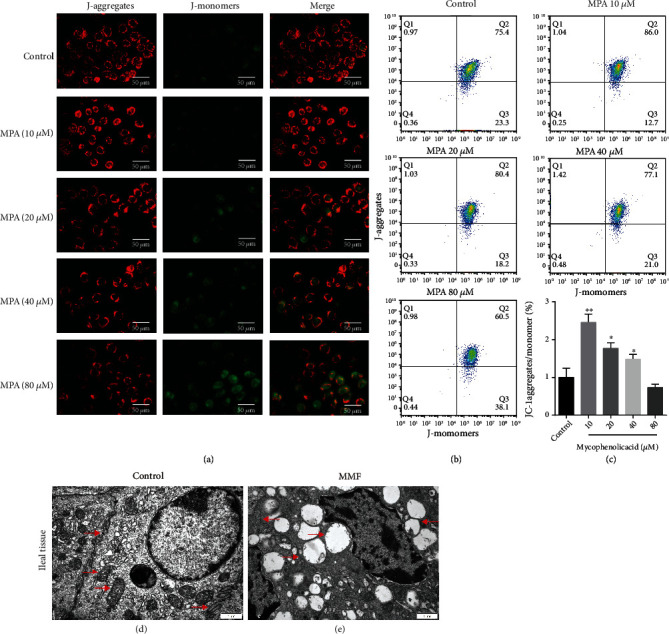
Effects of MPA on mitochondria. The ΔΨ_m_ depolarization was detected using fluorescence ((a) the bar represents 50 *μ*m) and flow cytometry (b, c) with JC-1 staining. MPA treatment resulted in ultrastructural changes of mitochondria (d, e) (the bar represents 1 *μ*m). Data are expressed as the means ± SD, *n* = 3; ^∗^*P* < 0.05, ^∗∗^*P* < 0.01 significantly different from the control.

It is well known that mitochondrial dysfunction is an important factor in elevated ROS and leads to apoptosis. The decrease in mitochondrial membrane potential (Δ*ψ*_m_) is often considered an indicator of mitochondrial dysfunction [18]. Therefore, to determine the changes in Δ*ψ*_m_, JC-1 probe was used for flow cytometry and fluorescence experiments. The probe forms JC-1 aggregates (red) at higher potentials and JC-1 monomers (green) at lower membrane potentials, and the ratio between red and green signals is a measure of Δ*ψ*_m_. Interestingly, after 24 h of MPA treatment of Caco-2 cells at 10 *μ*M, the cell distribution shifted toward a higher red-to-green ratio, indicating an increase in the membrane potential of at least some cells. However, as the MPA concentration increased from 20 to 80 *μ*M, the red fluorescence diminished and the green fluorescence enhanced, indicating that the mitochondrial membrane potential exhibited a trend of first increasing and then gradually decreasing (^∗^*P* < 0.05, ^∗∗^*P* < 0.01; Figures 6(a)–6(c)). In TEM observations, mitochondrial swelling and mitochondrial cristae structures were broken or even disappeared and with a large number of mitochondrial vacuoles in MMF-treated tissues compared to controls; tight junction structures were broken or widened, bridge grains disappeared, and the nuclei were crinkled, and chromatin was aggregated (red arrows, Figures 6 (d) and 6 (e)).

### 3.7. Effects of MPA on the Release of Cytochrome C and Bax Protein Expression

**Figure 7:**
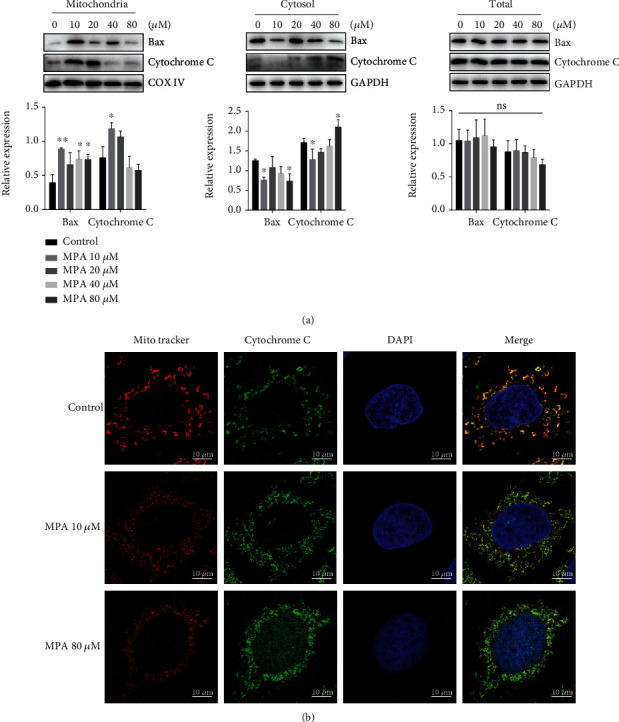
Effects of MPA on the release of Cytochrome C and Bax protein expression. Cytoplasmic (Cyto) and mitochondrial (Mito) fractions were prepared from Caco-2 cells treated with different concentrations of MPA for 24 h to analyze Cytochrome C and Bax protein expression (a). After treatment of Caco-2 cells with 10 *μ*M and 80 *μ*M MPA for 24 h, the distribution of Mito-tracker (red) and Cytochrome C (green) was examined by confocal microscopy (b). Data are expressed as the means ± SD, *n* = 3; ^∗^*P* < 0.05, ^∗∗^*P* < 0.01 significantly different from the control; the bar represents 10 *μ*m.

As shown in Figure 7(a), no significant elevation of Cytochrome C and Bax was detected in total cellular proteins after different concentrations of MPA treatment. Therefore, to further analyze the role of mitochondria in MPA-induced apoptosis, we examined the release of Cytochrome C and the transfer of Bax by separating mitochondrial and Cytoplasmic proteins for Western blot experiments. Compared with the control group, the protein level of Bax tended to decrease in the Caco-2 cytosol and increase in the mitochondria with increasing MPA concentrations. However, the protein level of Cytochrome C in mitochondria was significantly increased at 10 *μ*M, while Cytochrome C in the cytoplasm was decreased. When the dose of MPA was 80 *μ*M, the protein level of Cytochrome C in the solute of Caco-2 cells increased significantly, while that in the mitochondria decreased (Figure 7(a)). Mito-Tracker Red CMXRos is a recognized mitochondrial marker and we also observed the colocalization of Mito-Tracker Red CMXRos with Cytochrome C by fluorescence experiments. Similar to the Western blot results, Cytochrome C was concentrated in the mitochondria after 24 h exposure to 10 *μ*M MPA, while at 80 *μ*M, Cytochrome C was more distributed in the cytosol (Figure 7(b)).

### 3.8. Effect of Mitochondria-Targeted Antioxidant SS-31 on MPA-Induced Cytotoxicity

**Figure 8:**
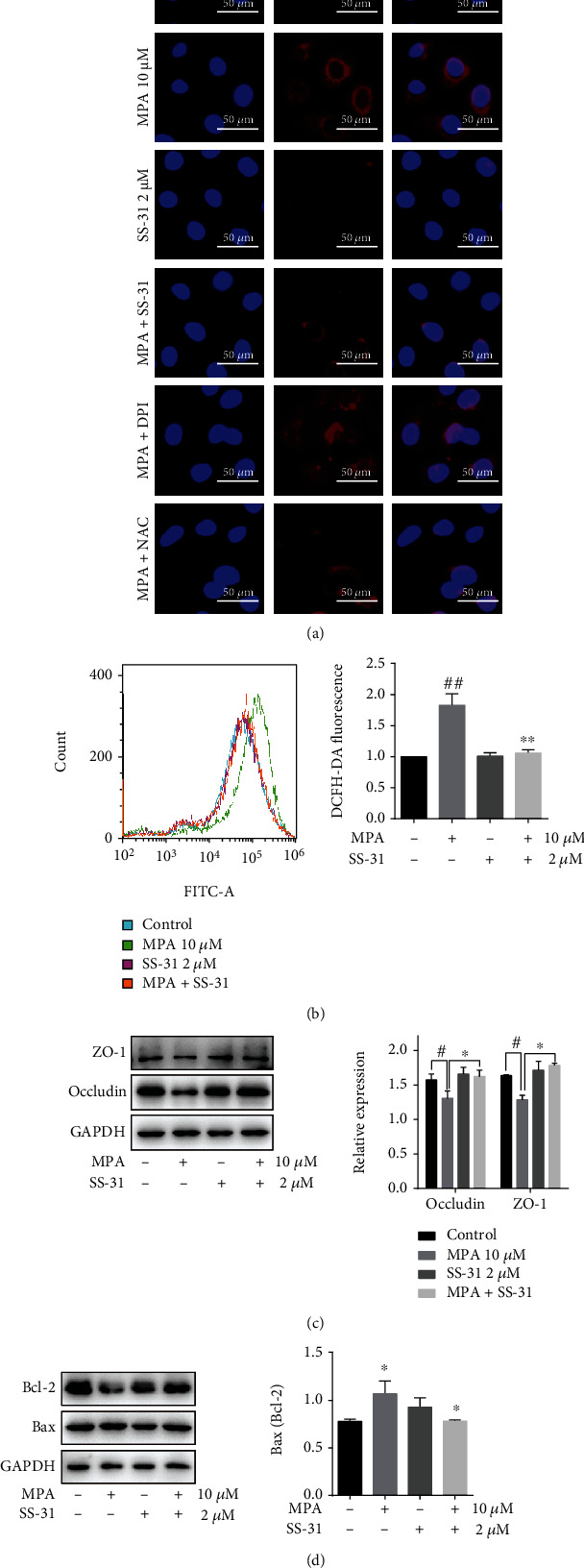
Effect of mitochondria-targeted antioxidant SS-31 on MPA-induced cytotoxicity. Effect of SS-31, NAC, and DPI on mitochondrial ROS in MPA-treated Caco-2 cells detected by MitoSOX staining (a). Effect of SS-31 on intracellular ROS detected by flow cytometry (b); protein expression of occludin, ZO-1 and Bax/Bcl-2 in Caco-2 cells assessed by Western blot (c, d). Data are expressed as the means ± SD, *n* = 3; ^∗^*P* < 0.05, ^∗∗^*P* < 0.01 significantly different from the control; ^#^*P* < 0.05, ^##^*P* < 0.01 significantly different from the mycophenolic acid-treated cells. The bar represents 50 *μ*m.

SS-31 is a mitochondria-targeted antioxidant that inhibits mitochondrial ROS production. By MitoSOX staining, we found that 2 *μ*M SS-31 pretreatment significantly reduced mitochondrial ROS levels in Caco-2 cells and NAC also decreased mitochondrial ROS, while DPI had no effect on this (Figure 8(a)). In addition, Caco-2 cells treated with SS-31 reduced the total intracellular ROS levels (Figure 8(b)) and restored occludin and ZO-1 protein expression at both protein and gene levels (Figure 8(c), Figure S2b) and reduced apoptosis (Figure 8(d)). These results suggest that SS-31 protects the intestinal mechanical barrier and reduces apoptosis *in vitro* by inhibiting MPA-induced oxidative stress.

## 4. Discussion

MPA inhibits immune rejection but its associated intestinal adverse effects severely limit its use [19]. Our results are consistent with previous reports that cell viability was reduced in a dose-dependent manner, tight junction proteins were down-regulated in a time-dependent manner, and apoptosis was increased in intestinal tissues or Caco-2 cells treated with MPA. Furthermore, DAO and ET, which are indicators of intestinal barrier permeability, are released into the bloodstream at a higher rate when the intestine is damaged [20]. MPA treatment was found to significantly increase the levels of DAO and ET in mouse serum, indicating that MPA induced the impairment of intestinal barrier function.

ROS causes intestinal barrier defects by downregulating TJ at all levels of the signaling pathways [21], and ROS-related apoptosis also inevitably impairs the tight junction structures between cells [22]. Current research has not determined which of these two mechanisms is more important for intestinal mechanical barrier dysfunction, or the exact sequence in which they occur. However, there is no doubt that either mechanism plays a significant role in the disruption of the intestinal epithelial barrier, resulting in increased intestinal permeability, which, in turn, triggers intestinal adverse effects such as diarrhea [23]. Therefore, we hypothesized that MPA downregulates TJ and induces apoptosis via ROS. The results suggest that MPA induces ROS production to promote oxidative stress, whereas the free radical scavenger NAC effectively upregulates TJ expression and reduces apoptosis.

There are multiple intracellular sources of ROS, of which NADPH oxidase, the main enzyme producing ROS, is a multicomponent enzyme complex found mainly in phagocytic and nonphagocytic cells. The activation of NADPH oxidase promotes the production of ROS [24]. However, the NOX 1 isoform, which is expressed in the intestine, showed no significant changes in protein level or enzyme activity in our experiments, while the expression level of its ligand, p22 protein, increased and then decreased over time. The use of DPI, an NADPH oxidase inhibitor, also failed to restore TJ damaged by MPA or reduce apoptosis and even caused cytotoxicity as DPI concentrations increased. These results suggest that MPA-induced oxidative stress may not be due to the activation of NADPH oxidase and the excessive inhibition of NADPH oxidase may disrupt its role in maintaining intestinal barrier stability. The mitochondria are another major source of ROS [25]. It has been shown that ROS produced by NADPH oxidase and ROS produced by mitochondria play different roles. ROS produced by NOXs affect cell proliferation and differentiation by regulating cell signaling [26], whereas excess ROS in the mitochondria induce apoptosis by increasing Bax expression [27, 28].

Depolarization of the mitochondrial membrane potential (ΔΨ_m_) is thought to be a critical step in the initiation of apoptotic signaling [29]. We first used the JC-1 dye to detect ΔΨ_m_ to investigate whether MPA promotes oxidative stress by damaging the mitochondria. Interestingly, the ratio of red to green fluorescence increased in the 10 *μ*M MPA-treated group compared to the control group, indicating that the ΔΨ_m_ did not decrease but rather increased, implying that the ΔΨ_m_ may be in a hyperpolarized state. It has been reported that hyperpolarization may be a prerequisite for mitochondria-mediated apoptotic death, which ultimately results in the loss of ΔΨ_m_ [30, 31]. Our experiments also revealed that the mitochondrial membrane potential increased and then decreased with increasing MPA concentration. Studies have shown that early mitochondrial hyperpolarization causes apoptosis [30]. By colocalization fluorescence experiments with Mito-Tracker and Western blot experiments isolating mitochondrial proteins, we found that apoptotic signals upregulate Cytochrome C in the cytoplasm and partially translocate it to the mitochondria, resulting in the accumulation of Cytochrome C in the mitochondria. As the concentration of MPA increases, Cytochrome C synthesis in the cytoplasm increases and continues to move to the mitochondria, while Cytochrome C is gradually released from the mitochondria to the cytoplasm and apoptotic cells eventually die [32]. Notably, the studies on Bax protein during mitochondrial membrane hyperpolarization have received less attention and our results indicate that Bax protein translocates to the mitochondria after treatment with different concentrations of MPA. We hypothesize that MPA promotes Bax translocation to the mitochondria and increases interaction with the permeability transition pore, thereby promoting the release of proteins like Cytochrome C and inducing apoptosis [33, 34]. To further determine whether the MPA-induced elevation in ROS was due to mitochondrial dysfunction, we used an inhibitor of Cytochrome C peroxidase, SS-31, which is thought to ameliorate mitochondrial dysfunction [35]. The results showed that intracellular and mitochondrial ROS in Caco-2 cells pretreated with SS-31 were significantly reduced, TJ damaged by MPA were restored, and the ratio of Bax to Bcl-2 protein expression was reduced. Furthermore, mitochondrial ROS were reduced significantly in the NAC group but not in the DPI group. These results suggest that the MPA-induced increase in ROS was due to mitochondrial dysfunction caused by damage to the mitochondria.

In conclusion, in the present study, we confirmed that the MPA-induced downregulation of occludin and ZO-1 expression, as well as apoptosis, in mice intestinal tissues and Caco-2 cells is mediated, at least in part, by the abnormal accumulation of ROS caused by mitochondrial dysfunction. Different concentrations of MPA produced different degrees of damage to mitochondria. At low concentrations, MPA induced a transient hyperpolarization of the ΔΨ_m_, and Cytochrome C accumulated in the mitochondria. As the concentration of MPA increased, the ΔΨ_m_ gradually decreased and Cytochrome C was released into the cytoplasm. The study provides the first evidence that mycophenolic acid-induced damage to the intestinal mechanical barrier is mediated by mitochondrial ROS.

However, there are some limitations to our study. Firstly, the mitochondrial effects on tight junctions were not explored in sufficient depth and the mechanism of MPA causing intestinal damage via mitochondria lacks more detailed in vivo experimental validation. Secondly, the reduced expression of several NADPH oxidases suggests that their role in MPA-related intestinal injury needs to be further clarified.

## 5. Conclusions

Our studies suggest that MPA induces intestinal toxicity *in vivo* and *in vitro*, at least in part, by impairing mitochondrial function and promoting oxidative stress to regulate TJ and apoptosis.

## Figures and Tables

**Table 1:** 

Genes	Forward primer (5′–3′)	Reverse primer (5′–3′)
GAPDH	CAAGGTCATCCATGACAACTTTG	GTCCACCACCCTGTTGCTGTAG
Occludin	ACAAGCGGTTTTATCCAGAGTC	GTCATCCACAGGCGAAGTTAAT
ZO-1	CAACATACAGTGACGCTTCACA	CACTATTGACGTTTCCCCACTC
NOX 1	GGAAACCGTGTCAGTCCTCC	AACCACTCACGACAACAAGTTTA
NOX 2	TTCCAGTGCGTGTTGCTCGAC	GATGGCGGTGTGCAGTGCTAT
NOX 4	GGATCACAGAAGGTCCCTAGCAG	GCGGCTACATGCACACCTGAGAA
p22phox	TGTGGTGAAGCTTTTCGGGC	GGATGGCTGCCAGCAGATAGAT
p67phox	CTATCTGGGCAAGCCTACGGTT	CACAAAGCCAAACAATACGCG

## Data Availability

The data used to support the findings of this study are available from the corresponding author upon request (Dr. Dujuan Xu, xudujuan2011@163.com).

## Abbreviations

Bax: Bcl-2-associated X protein
Bcl-2: B-cell lymphoma gene 2
Caspase 3: Cysteinyl aspartate-specific proteinase-3
DAO: Diamine oxidase
DPI: Diphenyleneiodonium
ET: Endotoxin
H&E: Hematoxylin-eosin staining
MDA: Malondialdehyde
MMF: Mycophenolate mofetil
MPA: Mycophenolic acid
NAC: N-Acetyl cysteine
NADPH: Nicotinamide adenine dinucleotide phosphate
NOX: NADPH oxidase
NOX 1: NADPH-OX-1
ROS: Reactive oxygen species
SOD: Superoxide dismutase
SS-31: D-Arg-Dmt-Lys-Phe-NH2
TJ: Tight junction
ZO-1: Zonulin occludin-1.

## Supplementary Materials

Supplementary Materials Figure S1: effects of MPA on NADPH oxidase. Figure S2: effect of different treatments on the mRNA expression levels of occludin, ZO-1, and NADPH oxidase-related genes. The Supplementary Material for this article can be found in the supplemental files.
