# Supplementary material for: Mycophenolic Acid Induces the Intestinal Epithelial Barrier Damage through Mitochondrial ROS
Source: Oxid Med Cell Longev. 2022 Jul 5;2022:4195699. doi: 10.1155/2022/4195699 (PMC9277164; doi:10.1155/2022/4195699)
Supplement: Supplementary Materials — Figure S1: effects of MPA on NADPH oxidase. Figure S2: effect of different treatments on the mRNA expression levels of occludin, ZO-1, and NADPH oxidase-related genes. The Supplementary Material for this article can be found in the supplemental files. [file 4195699.f1.docx]

**Supplemental Files**


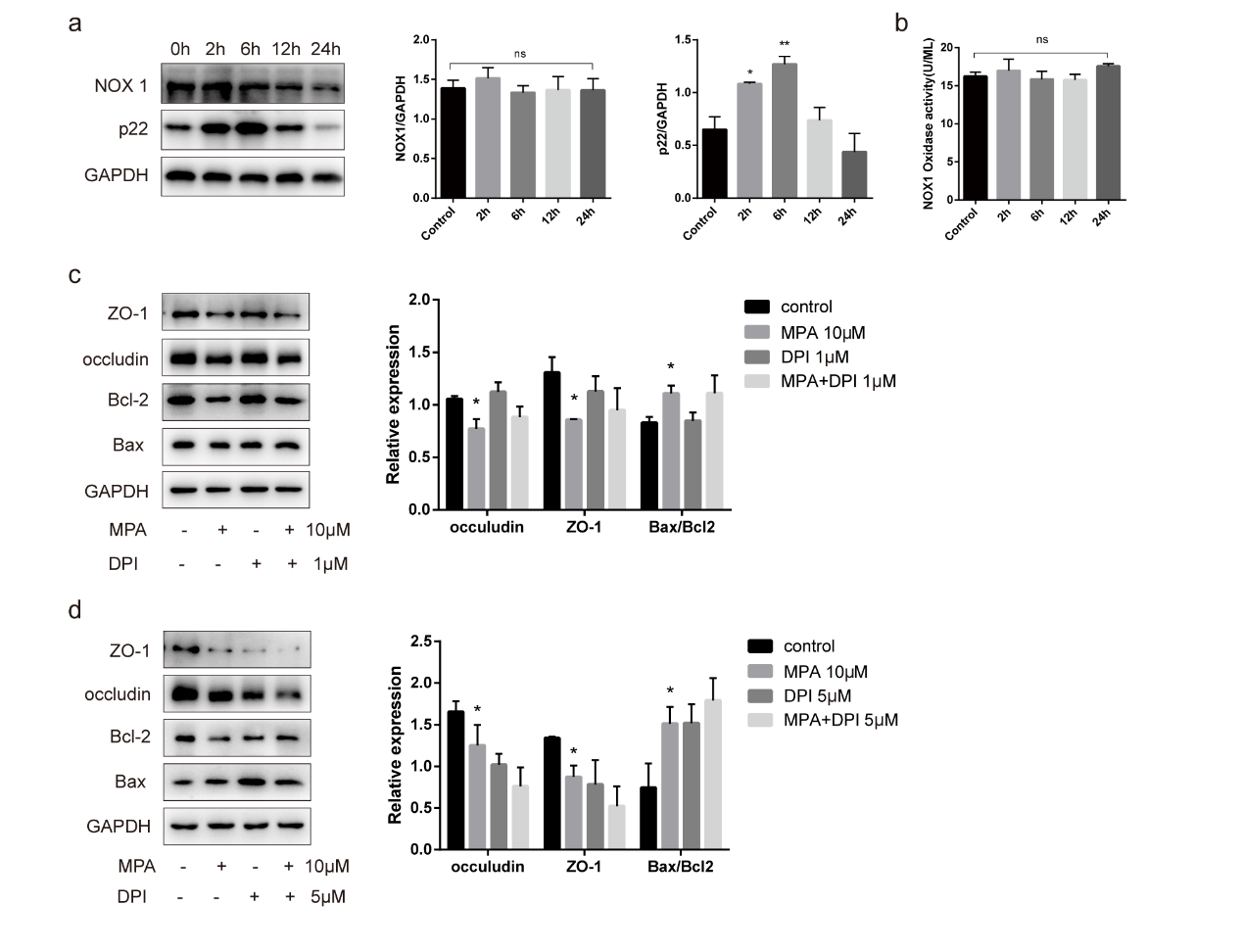


Figure S1. Effects of MPA on NADPH oxidase. Protein expression of NOX-1 and p22 in Caco-2 cells assessed by Western blot (a). Enzyme activity of NOX-1 in Caco-2 cells assessed by ELISA (b). Occludin, ZO-1 and Bax/Bcl-2 protein expression in Caco-2 cells was detected by Western blotting (c, d) after exposure to 1 µM or 5 µM of DPI and 10 µM MPA for 24h. Dates are expressed as the means ± SD, n = 3, ^*^*P* < 0.05, ^**^*P* < 0.01 significantly different from control.


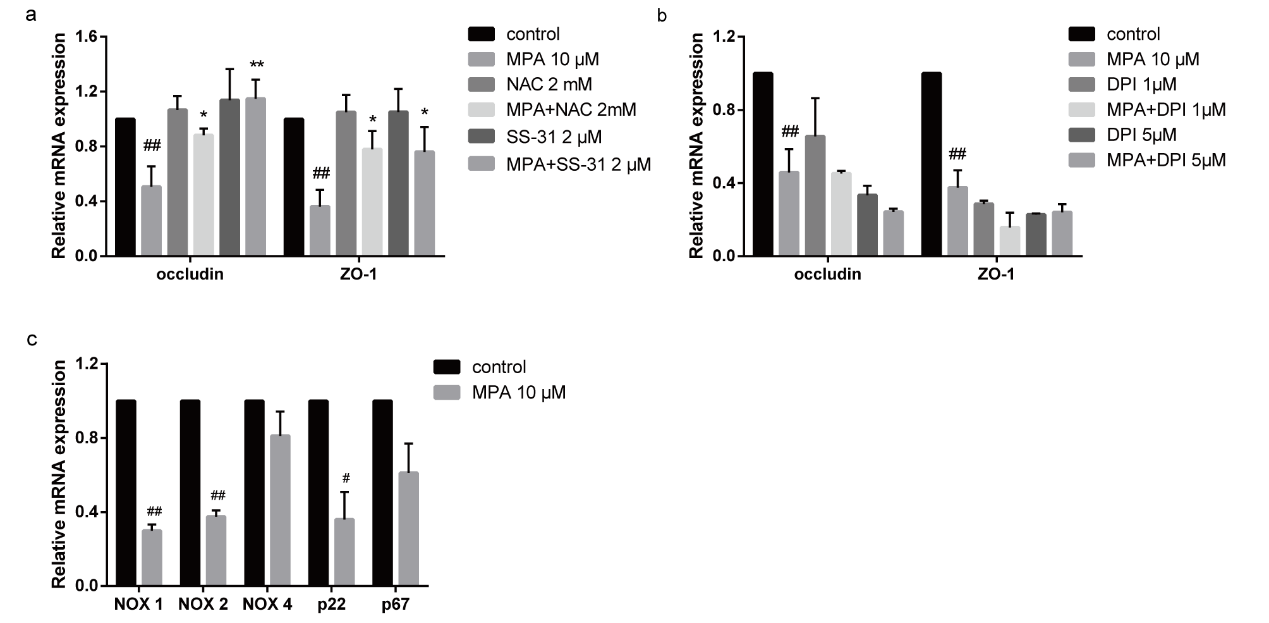


Figure S2. Effect of different treatments on the mRNA expression levels of occludin, ZO-1 and NADPH oxidase-related genes. qRT-PCR expression analysis of occludin and ZO-1 mRNA after addition of NAC, SS-31 (a) and DPI (b). qRT-PCR expression analysis of NADPH oxidase-related mRNAs (NOX 1, NOX 2, NOX 4, p22, p67) after MPA treatment (c). The fold change is calculated relative to the control group. ^#^*P* < 0.05, ^##^*P* < 0.01 significantly different from control. ^*^*P* < 0.05, ^**^*P* < 0.01 significantly different from control mycophenolic acid treated cells.
